# Overall Impact of the COVID-19 Pandemic on Interventional Radiology Services: A Canadian Perspective

**DOI:** 10.1177/0846537120951960

**Published:** 2020-08-30

**Authors:** Neeral R. Patel, Ghassan Awad El-Karim, Amol Mujoomdar, Sebastian Mafeld, Arash Jaberi, John R. Kachura, Kong Teng Tan, George D. Oreopoulos

**Affiliations:** 1 Division of Interventional Radiology, 7989 University Health Network, Toronto, Ontario, Canada; 2 Division of Interventional Radiology, 10033 London Health Sciences Centre, London, Ontario, Canada; 3 Division of Vascular Surgery, 7989 University Health Network, Toronto, Ontario, Canada

**Keywords:** COVID-19, pandemic, interventional radiology, service provision

## Abstract

**Purpose:**

The aim of this national survey was to assess the overall impact of the coronavirus disease 2019 (COVID-19) pandemic on the provision of interventional radiology (IR) services in Canada.

**Methods:**

An anonymous electronic survey was distributed via national and regional radiology societies, exploring (1) center information and staffing, (2) acute and on-call IR services, (3) elective IR services, (4) IR clinics, (5) multidisciplinary rounds, (6) IR training, (7) personal protection equipment (PPE), and departmental logistics.

**Results:**

Individual responses were received from 142 interventional radiologists across Canada (estimated 70% response rate). Nearly half of the participants (49.3%) reported an overall decrease in demand for acute IR services; on-call services were maintained at centers that routinely provide these services (99%). The majority of respondents (73.2%) were performing inpatient IR procedures at the bedside where possible. Most participants (88%) reported an overall decrease in elective IR services. Interventional radiology clinics and multidisciplinary rounds were predominately transitioned to virtual platforms. The vast majority of participants (93.7%) reported their center had disseminated an IR specific PPE policy; 73% reported a decrease in case volume for trainees by at least 25% and a proportion of trainees will either have a delay in starting their careers as IR attendings (24%) or fellowship training (35%).

**Conclusion:**

The COVID-19 pandemic has had a profound impact on IR services in Canada, particularly for elective cases. Many centers have utilized virtual platforms to provide multidisciplinary meetings, IR clinics, and training. Guidelines should be followed to ensure patient and staff safety while resuming IR services.

## Abstract (fr)

**Objectif:**

L’objectif de ce sondage national était d’évaluer l’effet global de la pandémie de coronavirus 2019 (COVID-19) sur les prestations de services de radiologie interventionnelle (RI) au Canada.

**Méthodes:**

Un sondage électronique anonyme a été distribué par le biais des sociétés de radiologie nationales et régionales pour examiner : (1) les informations sur les centres et le personnel, (2) les services d’urgence et de garde, (3) les services non urgents, (4) les cliniques de RI, (5) les équipes pluridisciplinaires, (6) les formations de RI, (7) les équipements de protection individuelle (ÉPI), et la logistique dans les différents départements.

**Résultats:**

Nous avons reçu des réponses individuelles de 142 radiologistes interventionnels au Canada (taux de réponse estimé à 70 %). Presque la moitié des participants (49,3 %) ont signalé une diminution générale des demandes de services de RI d’urgence; les services de garde étaient maintenus dans les centres fournissant habituellement ces services (99 %). La majorité des sondés (73,2 %) effectuaient les procédures de RI chez des patients hospitalisés et au chevet du patient lorsque possible. La plupart des sondés (88 %) ont rapporté une diminution générale des services de RI non urgents. Les cliniques de radiologie interventionnelle et les équipes pluridisciplinaires ont majoritairement migré vers des plateformes virtuelles. La vaste majorité des participants (93,7 %) ont indiqué que leur centre avait adopté une politique spécifique liée à l’utilisation d’ÉPI pour la radiologie interventionnelle; 73 % ont rapporté une diminution du nombre de cas d’au moins 25 % pour les stagiaires, et une partie des stagiaires verront leur début de carrière de radiologiste interventionnel (24 %) ou encore leur fellowship (35 %) retardés.

**Conclusion:**

La pandémie de COVID-19 a eu un effet significatif sur les services de radiologie interventionnelle au Canada, notamment pour les cas non urgents. De nombreux centres ont eu recours à des plateformes virtuelles pour les réunions pluridisciplinaires, les cliniques de RI et la formation. Lors de la reprise des services de RI, les directives doivent être respectées afin d’assurer la sécurité des patients et du personnel.

## Introduction

The novel human coronavirus disease 2019 (COVID-19) pandemic has created significant challenges for health care systems and disruptions in medical training globally.^1^ Health care organizations continue to reorient their services to prevent further spread of the disease with significant backlogs of rescheduled nonurgent or elective cases.

Guidelines have been created by the Society of Interventional Radiology and the Canadian Association of Interventional Radiologists to protect public safety and interventional radiology (IR) teams, as well as to optimize resource utilization while providing essential health care services.^2,3^ Specific recommendations have also been published with respect to balancing the interventional care of oncology patients against the risk of COVID-19.^4^ As the rate of COVID-19 cases begins to decline in Canada, plans must be implemented at the national and local level to ensure nonurgent and elective IR services are restarted in a safe manner. It is also important to learn where measures can be tightened or modified, given the possibility of a second or third wave of the pandemic.

The aim of this national survey was to assess the overall impact of the COVID-19 pandemic on the provision of IR services at both academic and community hospitals in Canada.

## Methods

This study conformed to the principles of the 1975 Declaration of Helsinki and was exempt by the institutional review board. An anonymous electronic survey was developed to broadly assess the impact of the COVID-19 pandemic on various aspects of IR service delivery and training in Canada. The survey was composed of 35 questions in total, split into 7 domains:

1. Centre information and staffing (7 questions),
2. Acute and on-call IR services (6 questions),
3. Elective IR services (defined as those procedures scheduled in advance, and not performed for acute or emergency indications; 6 questions),
4. IR clinics (2 questions),
5. Multidisciplinary rounds (2 questions),
6. IR training (6 questions), and
7. COVID-19 personal protection and logistics (6 questions).

The full questionnaire can be found in the Supplemental Material (Appendix A). The survey was uploaded to the Google Forms platform (Google, California, United States).

A standardized email outlining the purpose of the survey with an electronic link to the questionnaire was sent to all members of Canadian Association of Radiologists, Canadian Association of Interventional Radiologists, Ontario Association of Radiologists, Quebec Association of Radiologists, and Alberta Society of Radiologists. Members of the associations were asked to complete the survey if they were vascular and interventional radiologists (VIRs). Respondents who were not involved in VIR practice were excluded from the analysis. The survey was open for 25 days between May 5, 2020, and May 28, 2020. A reminder email was sent at the 2-week interval.

### Statistics

Categorical data were described as counts and percentages of respondents. Associations between categorical variables were assessed with Fisher exact test. Data collection was performed using Excel (Microsoft). Statistical analysis was performed using GraphPad Prism (GraphPad LLC). A *P* value of <.05 was deemed to be statistically significant.

## Results

### Demographics and Center Information

**Figure 1.**
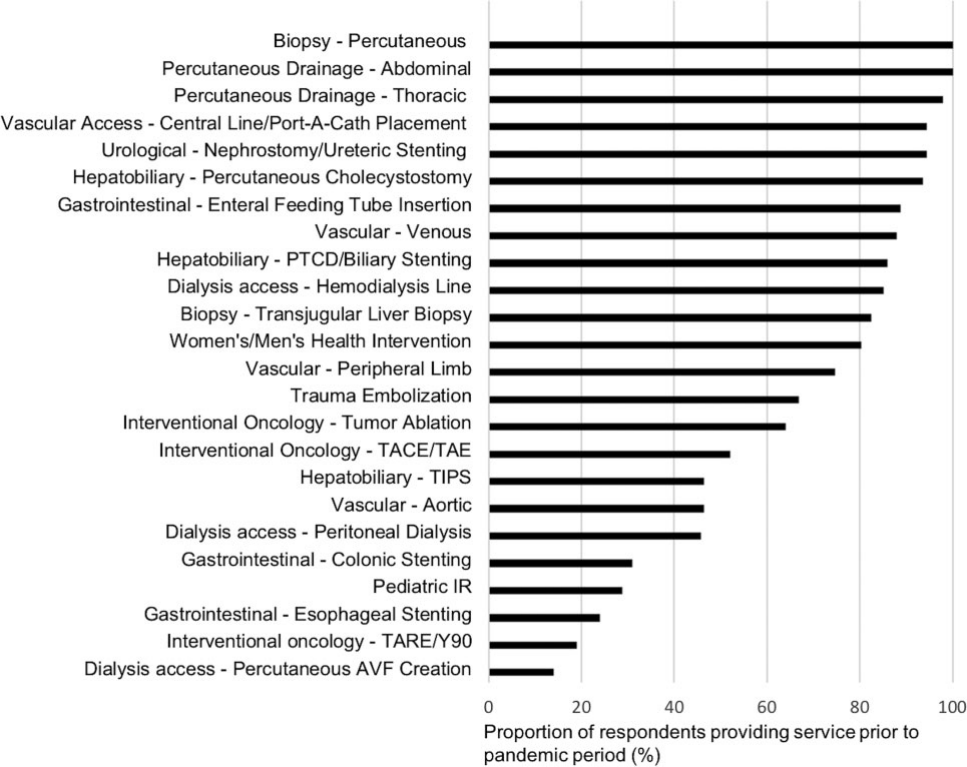
Summary of all services provided by interventional radiology departments by procedure in Canada.

A total of 145 responses were received, of which 142 were from VIRs and included in the analysis. Most recent Canadian Association for Interventional Radiology (CAIR) membership data for 2020 identified 166 IR staff and 37 IR fellows in training (203 total) in Canada. The response rate from IR respondents in Canada was therefore estimated at 142/203 (70.0%). Of the IR respondents, 78 (54.9%) were from Ontario, 31 (21.8%) from Quebec, 13 (9.3%) from British Columbia, 6 (4.2%) from Alberta, 4 (2.8%) from Nova Scotia, 3 (2.1%) from New Brunswick, 3 (2.1%) from Manitoba, 3 (2.1%) from Newfoundland and Labrador, and 1 (0.7%) from Saskatchewan. There was marginally more representation from tertiary care centers compared to community hospitals (56.3% and 43.7%, respectively). Interventional radiology represented greater than 50% of the clinical practice for 76 (54%) participants, 20% to 50% for 47 (33%) participants, and less than 25% for 19 (13%) participants. Figure 1 summarizes the IR services performed in the centers of respondents prior to the COVID-19 pandemic. The majority of participants (73.2%) routinely provided on-call IR services (71.2% in tertiary care centers and 28.8% in community hospitals).

### Impact on Acute and On-Call IR Services

**Figure 2.**
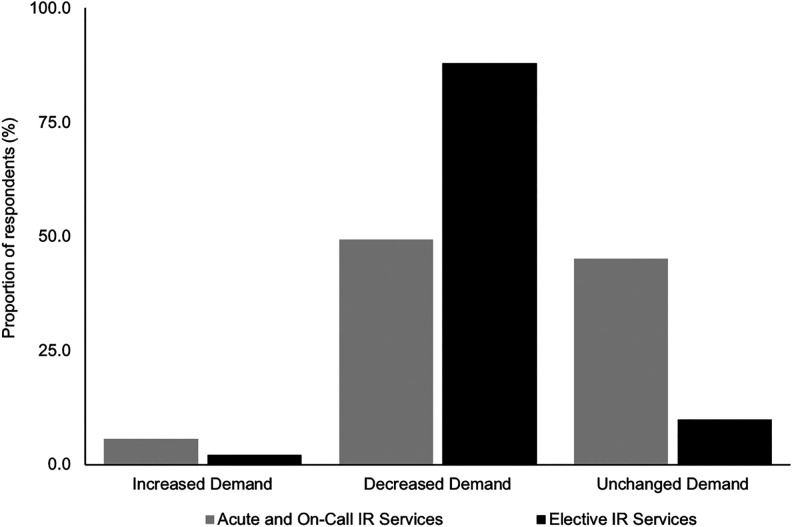
Bar graph summarizing reported demand for acute and elective interventional radiology services during coronavirus disease 2019 (COVD-19) pandemic.

Nearly half of the participants (49.3%) reported an overall decrease in demand for acute IR services (Figure 2), while only 8 (5.6%) participants reported an increase in demand. Percutaneous thoracic drainage (10.3%), percutaneous abdominal drainage (7.5%), nephrostomy/ureteric stent insertion (7.5%), venous interventions (5.5%), and percutaneous cholecystostomy (4.1%) were identified as the acute IR services with the highest demand during the pandemic.

Of the 104 respondents who routinely provide on-call IR services, 29% stated that they had to modify their normal IR rota (on-call and/or daytime) in order to continue to provide on-call services; 1 participant reported they were not able to provide an on-call IR service.

### Impact on Elective IR Services

**Figure 3.**
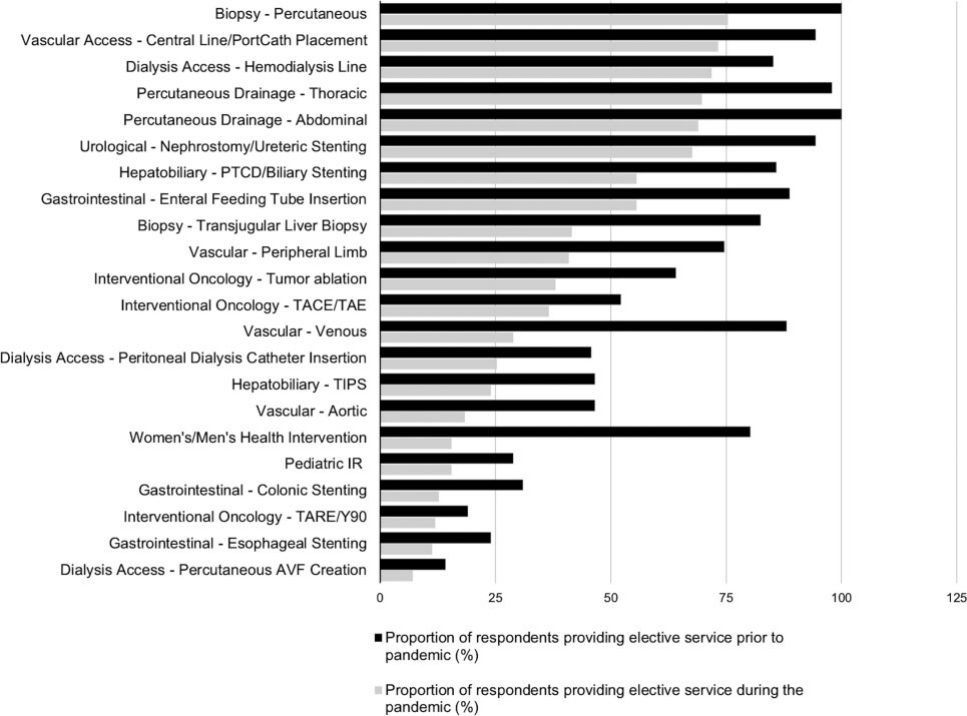
Summary of all elective interventional radiology services for which provision was maintained during coronavirus disease 2019 (COVID-19) pandemic (grey bars) relative to service provision prior to the pandemic (black bars).

The majority of participants (88%) reported an overall decrease in elective IR services (Figure 2). Elective procedures with the least demand during COVID-19 included women’s/men’s health interventions (40.4%), peripheral limb vascular procedures (36.3%), venous vascular procedures (28.8%), transjugular liver biopsies (24.7%), and aortic vascular procedures (22.6%). Elective procedures with a reported increased demand during COVID-19 included gastrointestinal feeding tubes (7.5%), nephrostomy/ureteric stenting (7.5%), percutaneous abdominal drainage (6.2%), peritoneal dialysis catheter insertion (6.2%), and hemodialysis catheter insertion (5.5%). Figure 3 summarizes the elective IR services which continued to be performed during the pandemic.

Most of the participants (88.4%) with access to a day case recovery unit stated that it remained fully functional during the pandemic, while 11.6% reported that their unit was either nonfunctional or at reduced capacity.

### Impact on Clinic Visits and Multidisciplinary Rounds

Most respondents (75.4%) had to modify or cancel their IR clinics as a result of the pandemic with 48.6% moving to a virtual platform, 29% triaging referrals and reviewing those eligible in-person, and 22.4% canceling their clinics completely. Similarly, the majority of participants (81.7%) reported that they had discontinued face-to-face multidisciplinary rounds and instead predominately used videoconferencing (96.6%) to continue to conduct multidisciplinary discussions.

### Personal Protection and Departmental Logistics

The vast majority of participants (93.7%) reported their center had disseminated an IR-specific personal protective equipment (PPE) policy to members of the department, and that they were readily able to follow this policy. Most (85.2%) reported some form of modification to working patterns, with the majority of respondents splitting into 2 teams working on alternate weeks (75.9%). Only 12.4% were asked to support other specialties, and 11.7% had a backup on-call rota in anticipation of possible illness or quarantine of IR attendings.

Most respondents (73.2%) were performing procedures at the patient bedside for COVID suspected or positive patients when possible. Only 31.7% were providing a dedicated portable central venous line service for patients with COVID-19, and 33% were routinely testing elective/semiurgent outpatients for COVID-19 prior to scheduled procedures. There was no statistical difference between participants from tertiary or community hospitals regarding these responses.

More than half of participants (65.5%) did not experience significant staff shortages as a result of the pandemic. Of respondents who reported significant shortages, these were related to technologists (31%), IR attendings (24%), nursing staff (22%), trainees (12%), and porters (11%). Shortages were secondary to self-isolation (42.9%), forced vacation time (27.6%), redeployment (17%), and child care (12.5%). Eleven (7.7%) participants reported a COVID-19 outbreak at their institution.

### Impact on Training

Less than half of the participants (40.8%) reported providing IR fellowship training at their centers. Of those who did, the vast majority (89.7%) reported a decrease in the average case-load volume for trainees; 73% reported a decrease in daily case volume for trainees by at least 25%. Most respondents (89.7%) reported that their trainees have not been redeployed to other specialties. With respect to supplemental education, only 25.9% of respondents had arranged virtual trainee teaching.

While the majority of participants reported that COVID-19 did not have an impact on the future employment positions for current IR trainees (69%) or the positions for incoming fellows (60%), a considerable percentage of trainees will either be delayed to start their careers as IR attendings (24%) or fellowship training (35%); some had lost their future arrangements as IR attendings (7%) or fellowship positions (5%).

## Discussion

The COVID-19 pandemic has resulted in major disruptions to radiology services across Canada. Recent surveys administered by the Canadian Association of Radiologists and the Canadian Association of Medical Radiation Technologists have shown an overall 50% to 70% drop in radiology services.^5^ The results of this survey provide the first snapshot of the impact of COVID-19 specific to the multiple facets of IR service provision in Canada.

### Acute IR Services

Approximately 50% of all respondents reported a decreased demand for acute IR services. This correlates with a widespread reduction in emergency department (ED) attendances for non-COVID related presentations in both adult and pediatric populations due to fears of contracting the virus. One Italian study estimated a substantial fall in pediatric ED admissions by 73% to 88% during the pandemic in comparison with the preceding year.^6^ The consequence of fewer ED admissions is the delayed presentation of common surgical emergencies such as acute appendicitis, diverticulitis, and cholecystitis; in cases of delayed presentation, when patients do attend the ED they are likely to have complications of these conditions, including perforation with abscess formation requiring image-guided intraabdominal drainage by IR. Furthermore, surgical lists were culled to create capacity for a surge in COVID-19 admissions, limit PPE consumption given the uncertainty about adequacy of supply, and also to limit risk of viral spread through aerosol generating procedures such as induction of general anesthesia and endoscopy. As a consequence, the demand for temporizing minimally invasive measures such as nephrostomy and percutaneous cholecystostomy tube insertion increased as evidenced by this survey.

The survey also demonstrated demand for venous IR procedures had increased during the pandemic. Studies have noted a significant increased risk of venous thromboembolism in patients diagnosed with COVID-19, particularly those admitted to the intensive care unit (ICU).^7,8^ One theory is that the increased demand for venous interventions may have been due to a greater number of thrombolysis procedures performed for deep venous thrombosis or pulmonary embolism, although there is currently no high quality data in the literature to support this.^9^ The increased sedentary lifestyle of the population during the pandemic may be another explanation with regard to the increased incidence of venous thromboembolism during the pandemic.^10,11^

Fortunately, the majority of IR departments that were involved in the survey were able to maintain the after-hours call service. It is encouraging that a high proportion of respondents (73.2%) were performing acute procedures at the patient bedside where possible, thereby reducing the risk of transmission within the respective hospitals.

### Elective IR Services

The vast majority of respondents (88%) reported a decrease in provision of elective services. The areas most impacted included women’s and men’s health interventions, peripheral vascular interventions, and aortic interventions. The reduced provision of elective service by IR was a necessary intervention during the pandemic to ensure minimal risk to patients and staff with respect to virus transmission. Procedures for which a delay would not lead to a significant reduction in quality of life or outcomes were required to be postponed by IR departments in accordance with national and international guidance.^2,3^ The potential impact of delayed care for other conditions including abdominal aortic aneurysms for endovascular repair and endovascular reconstruction for patients with peripheral arterial disease and critical limb threatening ischemia remains to be defined. Some services may ultimately see a “surge” in patients with non-COVID conditions with more advanced disease presentations secondary to COVID-19 related delays in accessing care.

Participants who reported increased elective provision stated these were primarily in gastrostomy feeding tube insertion and dialysis access. As endoscopy provision plummeted during the pandemic period, combined with a rise in enteral feeding requirements for patients with COVID-19 on ICU, greater numbers of radiologic gastrostomy tubes were likely to have been performed in certain regions. For patients requiring dialysis, a reduction in surgical provision of arteriovenous access creation also lead to an increased demand for dialysis catheter or peritoneal dialysis catheter insertion in some centers. As operating rooms around the country gradually return to normal operations, there will be a backlog of oncology cases that are expected to take a priority, and elective arteriovenous access procedures may continue to be delayed. This raises the question as to whether or not the creation of percutaneous arteriovenous fistulae deserves an expanded role in the angiography suite to cover this potential care gap.

The Canadian Association of Radiologists have recently published a report of principles and general guidelines to facilitate resumption of radiology services.^5^ The public health response to COVID-19 has resulted in a 50% to 70% decrease in radiology services from March to April 2020, and a significant backlog of postponed and rescheduled cases is expected. In order to facilitate an effective resumption of radiology services, the report outlines 5 ways to modify practices: (1) setup a radiology task force; (2) triage, categorize, and segregate patients of varying risks; (3) ensure adequate human resources to deal with the crisis; (4) minimize unnecessary imaging for suspected or confirmed patients with COVID-19, and (5) continue to advocate for workplace and social responsibility. With respect to IR, procedures must be triaged according to the procedural type and clinical indication. The Society of Interventional Radiology has created a practical framework with an algorithm to plan for resuming IR services according to clinical priority.^12^

Although cutting through the inevitable backlog of cases will be of importance for many departments, ensuring this is done in a safe manner for both staff and patients is essential; 33% of respondents stated patients are being tested for COVID-19 prior to routine or semielective cases. This figure is below expectations but may be explained by the low capacity for testing which was widespread at the start of the pandemic, with a subsequent rapid increase in testing capability more recently.^13^ The Ontario Ministry of Health has published guidance with respect to recommencing surgeries and procedures during the pandemic and state for areas where there is a high rate of community transmission patients should undergo testing 24 to 48 hours prior to a procedure requiring general anesthetic. Considering most IR procedures will be performed without general anesthetic, consensus is required with regard to testing, particularly prior to aerosol generating procedures. It is reassuring that the majority of respondents reported adequate access to PPE, however for those in centers where there is a shortage of PPE, staff should not be put at risk with respect to restarting services, and PPE shortages in these centers should be addressed as a matter of urgency.

### Clinics and Multidisciplinary Rounds

The majority of participants stated they were able to continue multidisciplinary rounds and IR clinics with modifications which included the utilization of telemedicine tools allowing continuity of care during the height of the pandemic. One area of concern was that 29% of respondents stated they continued to perform face-to-face clinics after triaging, while 22.4% cancelled clinics altogether. The rise in availability of telemedicine software which is suitable for patient care should negate the requirement for face-to-face interactions during this pandemic and allow follow-up clinic visits to continue in most centers; greater uptake of telemedicine technologies by IR departments is therefore recommended to facilitate continuity of care.^14^

### Education and Training

The COVID-19 pandemic has resulted in profound changes across all levels of medical education and training. In Ontario, numerous clinical residents were redeployed to COVID-19 assessment units, EDs, internal medicine wards, and ICUs at both tertiary and community hospitals. Licensing examinations for final year residents were postponed until autumn 2020, and the objective structure clinical examinations cancelled.

The pandemic has also impacted diagnostic radiology and IR training in unprecedented ways.^1,15^ The IR fellows have seen a decrease in their case volumes and a shift in the types of procedures performed. Similarly, in this study, of the respondents who provide IR training, the vast majority reported a decrease in the volume for trainees, with 73% reporting a decrease in volumes by at least 25%. In Canada, competency decisions for IR trainees are at the discretion of individual fellowship programs. Interestingly, only 26% of respondents with trainees have created virtual trainee teaching. This is certainly an area for improvement given the easily accessible online platforms. Unfortunately, the survey also showed disruptions and even cancelations with respect to future employment positions for current IR trainees and incoming fellows. Safety nets should be considered at the program level to ensure smooth transition for these individuals, the most practical of which would be to extend the duration of fellowship training, particularly for those trainees most affected by a reduction in IR exposure as a result of the pandemic.

The upcoming 2021 Canadian Resident Matching Service residency program match is expected to have a more compressed timeline, with interviews being conducted in a virtual format, and it is expected that elective experiences will not be allowed to factor into program selection decisions as many students’ elective experiences were cancelled and will remain less accessible because of COVID-19 as we move into the next academic year in July. These changes may affect the nature and type of candidate applying to diagnostic radiology programs and the potential future IR applicant pool. The IR fellowship and residency programs, as well as CAIR, will need to become more flexible and proactive in terms of reaching out to medical students and residents to establish (virtual) mentoring and educational opportunities that will continue to promote an interest in pursuing IR as a career.

Travel restrictions have contributed to the cancellation of international and national IR conferences. Flight restrictions also resulted in difficulties for international trainees in departing from Canada after completion of fellowship, as well as for those entering the country prior to commencing fellowship.

Limitations of this study include responses being subjective in nature rather than objective, a disadvantage intrinsic to survey-related research. Changes in service provision identified were not based on documented data but rather the individual perception of responders. Responses may have also been skewed by those centers staffed by more interventional radiologists given there were no limitations on survey respondents by institution.

In conclusion, results from this national survey provide an in-depth overview of the impact of COVID-19 on IR services in Canada. In order to ensure a safe and effective resumption of radiology services, principles and general guidelines recently published by the Canadian Association of Radiologists and Society for Interventional Radiology should be reviewed and acted upon by all IR departments.

## Supplemental Material

Supplemental Material, Appendix_A_COVID-19_Survey - Overall Impact of the COVID-19 Pandemic on Interventional Radiology Services: A Canadian Perspective Click here for additional data file. Supplemental Material, Appendix_A_COVID-19_Survey for Overall Impact of the COVID-19 Pandemic on Interventional Radiology Services: A Canadian Perspective by Neeral R. Patel, Ghassan Awad El-Karim, Amol Mujoomdar, Sebastian Mafeld, Arash Jaberi, John R. Kachura, Kong Teng Tan and George D. Oreopoulos in Canadian Association of Radiologists Journal

## References

[1] Warhadpande S Khaja MS Sabri SS The impact of COVID-19 on interventional radiology training programs: what you need to know. Acad Radiol. 2020;27(6):868–871. 32359819 10.1016/j.acra.2020.04.024 PMC7183945

[2] Mujoomdar A Graham T Baerlocher MO Soulez G The Canadian Association for Interventional Radiology (CAIR) and Canadian Association of Radiologists (CAR) guidelines for interventional radiology procedures for patients with suspected or confirmed COVID-19. Can Assoc Radiol J. 2020;846537120924310. 32380846 10.1177/0846537120924310

[3] Society of Interventional Radiology - COVID-19 Clinical Notification. Published 2020 Accessed June 21, 2020 https://www.sirweb.org/practice-resources/covid-19-resources/covid-19-clinical-notification/

[4] Denys A Guiu B Chevallier P Digklia A de Kerviler E de Baere T Interventional oncology at the time of COVID-19 pandemic: problems and solutions. Diagn Interv Imaging. 2020;101(6):347–353. 32360351 10.1016/j.diii.2020.04.005 PMC7177103

[5] Canadian Association of Radiologists: Radiology Resumption of Clinical Services. Published 2020. Accessed June 11, 2020 https://car.ca/wp-content/uploads/2020/05/CAR-Radiology-Resumption-of-Clinical-Services-Report_FINAL.pdf

[6] Lazzerini M Barbi E Apicella A Marchetti F Cardinale F Trobia G Delayed access or provision of care in Italy resulting from fear of COVID-19. Lancet Child Adolesc Health. 2020;4(5):e10–11. 32278365 10.1016/S2352-4642(20)30108-5 PMC7146704

[7] Zhang L Feng X Zhang D, et al. Deep vein thrombosis in hospitalized patients with coronavirus disease 2019 (COVID-19) in Wuhan, China: prevalence, risk factors, and outcome. Circulation*.* 2020;142(2):114–128. 32421381 10.1161/CIRCULATIONAHA.120.046702

[8] Middeldorp S Coppens M van Haaps TF, et al. Incidence of venous thromboembolism in hospitalized patients with COVID-19. J Thromb Haemost. 2020;18(8):1995–2002. 32369666 10.1111/jth.14888 PMC7497052

[9] Marone EM Rinaldi LF Upsurge of deep venous thrombosis in patients affected by COVID-19: preliminary data and possible explanations. J Vasc Surg Venous Lymphat Disord. 2020;8(4):694–695. 32305586 10.1016/j.jvsv.2020.04.004 PMC7162769

[10] Kunutsor SK Mäkikallio TH Seidu S, et al. Physical activity and risk of venous thromboembolism: systematic review and meta-analysis of prospective cohort studies. Eur J Epidemiol. 2020;35(5):431–442. 31728878 10.1007/s10654-019-00579-2 PMC7250794

[11] Deschasaux-Tanguy M Druesne-Pecollo N Esseddik Y, et al. Diet and physical activity during the COVID-19 lockdown period (March-May 2020): results from the French NutriNet-Sante cohort study. medRxiv. (Pre-print). 2020. 10.1093/ajcn/nqaa336 PMC7989637 33675635

[12] Society of Interventional Radiology - Covid-19 postponed procedures. Published 2020 Accessed June 21, 2020 https://www.sirweb.org/practice-resources/toolkits/covid-19-toolkit/covid-19-postponed-procedures/

[13] Al-Muharraqi MA Testing recommendation for COVID-19 (SARS-CoV-2) in patients planned for surgery - continuing the service and ‘suppressing’ the pandemic. Br J Oral Maxillofac Surg. 2020;58(5):503–505. 32307131 10.1016/j.bjoms.2020.04.014 PMC7152878

[14] Punia V Nasr G Zagorski V, et al. Evidence of a rapid shift in outpatient practice during the COVID-19 pandemic using telemedicine. Telemed J E Health. 2020 doi:10.1089/tmj.2020.0150. 10.1089/tmj.2020.0150 32429769

[15] Alvin MD George E Deng F Warhadpande S Lee SI The Impact of COVID-19 on radiology trainees. Radiology. 2020;296(2):246–248. 32216719 10.1148/radiol.2020201222 PMC7233407

