# Supplementary material for: Overall Impact of the COVID-19 Pandemic on Interventional Radiology Services: A Canadian Perspective
Source: Can Assoc Radiol J. 2020 Aug 30:0846537120951960. doi: 10.1177/0846537120951960 (PMC7459179; doi:10.1177/0846537120951960)
Supplement: Supplemental Material, Appendix_A_COVID-19_Survey - Overall Impact of the COVID-19 Pandemic on Interventional Radiology Services: A Canadian Perspective [file Appendix_A_COVID-19_Survey.pdf]

COVID-19 Impact on Interventional Radiology Services in Canada

Thank you for taking the time to complete this survey assessing the impact of the COVID-19 pandemic on Interventional Radiology services in Canada. The survey will take between 5-10 minutes to complete. All information provided is anonymous.

* Required

Skip to question 1Skip to question 1

Section A: Centre Information and Staffing

1. 1. What proportion of clinical practice does Interventional Radiology represent on average for Interventional Radiologists at your centre? *

Mark only one oval.

- ( ) <25%
- ( ) 25-50%
- ( ) >50%
- ( ) No Interventional Radiology at our centre

2. 2. Which province/territory is your hospital based in? *

Mark only one oval.

- ( ) Alberta
- ( ) British Columbia
- ( ) Manitoba
- ( ) New Brunswick
- ( ) Newfoundland and Labrador
- ( ) Northwest Territories
- ( ) Nova Scotia
- ( ) Nunavut
- ( ) Ontario
- ( ) Prince Edward Island
- ( ) Quebec
- ( ) Saskatchewan
- ( ) Yukon

3. 3. How would you describe your centre? *

Mark only one oval.

- ( ) Tertiary referral centre
- ( ) Community hospital
- ( ) Other: ____________________

4. 4. What services does your IR department offer? *

Check all that apply.

- [ ] Vascular - Aortic
- [ ] Vascular - Peripheral Limb
- [ ] Vascular - Venous
- [ ] Trauma embolization
- [ ] Interventional oncology - Tumor ablation
- [ ] Interventional oncology - TACE/TAE
- [ ] Interventional oncology - TARE/Y90
- [ ] Dialysis access - Hemodialysis line and permanent access maintenance procedures
- [ ] Dialysis access - Peritoneal dialysis catheter insertion
- [ ] Dialysis access - Percutaneous AVF creation
- [ ] Hepatobiliary - PTCD/biliary stenting
- [ ] Hepatobiliary - TIPS
- [ ] Hepatobiliary - Percutaneous cholecystostomy
- [ ] Gastrointestinal - Enteral feeding tube insertion
- [ ] Gastrointestinal - Esophageal stenting
- [ ] Gastrointestinal - Colonic stenting
- [ ] Urological - Nephrostomy/ureteric stenting
- [ ] Women's/Men's health intervention - UFE/PAE/varicocele/pelvic congestion
- [ ] Percutaneous drainage - Thoracic
- [ ] Percutaneous drainage - Abdominal
- [ ] Vascular access - Central line/Portacath placement
- [ ] Biopsy - Percutaneous
- [ ] Biopsy - Transjugular liver biopsy
- [ ] Pediatric IR

Other: [ ] ____________________

5. 5. Have you modified the working pattern for attendings/faculty within your unit? Please tick all that apply. *

Check all that apply.

- [ ] No modifications
- [ ] Attendings asked to support other specialties
- [ ] Doubled up on-call/shadow on-call
- [ ] Reduced number of attendings in hospital

Other: [ ] ______________________

6. 6. Have significant staff shortages occurred in your IR department as a result of this pandemic? Please tick all that apply. *

Check all that apply.

- [ ] Yes, nursing staff
- [ ] Yes, technologists
- [ ] Yes, porter staff
- [ ] Yes, IR attendings
- [ ] Yes, IR trainees
- [ ] No significant staff shortages

7. 7. If you answered 'yes' to question 6, what are shortages of IR team members the result of? Please tick all that apply.

Check all that apply.

- [ ] Need for individuals to self-isolate/quarantine because of COVID-19
- [ ] Redeployment of team members to other areas within the hospital
- [ ] Redeployment of individuals to other areas outside of the hospital
- [ ] Increased numbers of 'sick days' taken by staff during the pandemic
- [ ] Increased need for team members to stay at home and care for children home from school
- [ ] Forced vacation time by your practice group

Skip to question 8

Section B: Acute and On-Call IR Service

8. 8. Overall has the demand for acute IR services changed? *

Mark only one oval.

( ) Yes, increased
( ) Yes, decreased
( ) No, unchanged

9. 9. In which areas have there been an increased demand for acute IR services, if any? Please tick all that apply. *

Check all that apply.

- [ ] None
- [ ] Vascular - Aortic
- [ ] Vascular - Peripheral Limb
- [ ] Vascular - Venous
- [ ] Trauma embolization
- [ ] Hepatobiliary - PTCD/biliary stenting
- [ ] Hepatobiliary - TIPS
- [ ] Hepatobiliary - Percutaneous cholecystostomy
- [ ] Urological - Nephrostomy/ureteric stenting
- [ ] Percutaneous drainage - Thoracic
- [ ] Percutaneous drainage - Abdominal
- [ ] Pediatric IR

Other: [ ] ______________________

10. 10. In which areas have there been a decreased demand for acute IR services, if any? Please tick all that apply. *

Check all that apply.

- [ ] None
- [ ] Vascular - Aortic
- [ ] Vascular - Peripheral Limb
- [ ] Vascular - Venous
- [ ] Trauma embolization
- [ ] Hepatobiliary - PTCD/biliary stenting
- [ ] Hepatobiliary - TIPS
- [ ] Hepatobiliary - Percutaneous cholecystostomy
- [ ] Urological - Nephrostomy/ureteric stenting
- [ ] Percutaneous drainage - Thoracic
- [ ] Percutaneous drainage - Abdominal
- [ ] Pediatric IR

Other: [ ] ______________________

11. 11. Do Interventional Radiologists at your centre routinely provide a 24/7 on-call service? *

Mark only one oval.

- Yes
- No

12. 12. If yes to question 11, are they still able to provide 24/7 on-call IR services currently?

Mark only one oval.

- Yes
- No
- Other: ______________________

13. 13. If yes to question 12, has there been any modification to the normal IR rota (on-call and/or daytime) to continue to provide the 24/7 cover?

Mark only one oval.

- Yes
- No
- Other: ______________________

Skip to question 14

Section C: Elective IR Service

14. 14. Overall, has the provision of elective IR services changed? *

Mark only one oval.

- Yes, increased
- Yes, decreased
- No, unchanged

15. 15. Which elective IR services have been maintained? Please tick all that apply. *

Check all that apply.

- [ ] Vascular - Aortic
- [ ] Vascular - Peripheral Limb
- [ ] Vascular - Venous
- [ ] Interventional oncology - Tumor ablation
- [ ] Interventional oncology - TACE/TAE
- [ ] Interventional oncology - TARE/Y90
- [ ] Dialysis access - Hemodialysis line and permanent access maintenance procedures
- [ ] Dialysis access - Peritoneal dialysis catheter insertion
- [ ] Dialysis access - Percutaneous AVF creation
- [ ] Hepatobiliary - PTCD/biliary stenting
- [ ] Hepatobiliary - TIPS
- [ ] Gastrointestinal - Enteral feeding tube insertion
- [ ] Gastrointestinal - Esophageal stenting
- [ ] Gastrointestinal - Colonic stenting
- [ ] Urological - Nephrostomy/ureteric stenting
- [ ] Women's/Men's health intervention - UFE/PAE/varicocele/pelvic congestion
- [ ] Percutaneous drainage - Thoracic
- [ ] Percutaneous drainage - Abdominal
- [ ] Vascular access - Central line/Portacath placement
- [ ] Biopsy - Percutaneous
- [ ] Biopsy - Transjugular liver biopsy
- [ ] Pediatric IR

Other: [ ] ______________________

16. 16. In which areas have you seen an increased demand for elective IR services? Please tick all that apply. *

Check all that apply.

- [ ] None
- [ ] Vascular - Aortic
- [ ] Vascular - Peripheral Limb
- [ ] Vascular - Venous
- [ ] Interventional oncology - Tumor ablation
- [ ] Interventional oncology - TACE/TAE
- [ ] Interventional oncology - TARE/Y90
- [ ] Dialysis access - Hemodialysis line and permanent access maintenance procedures
- [ ] Dialysis access - Peritoneal dialysis catheter insertion
- [ ] Dialysis access - Percutaneous AVF creation
- [ ] Hepatobiliary - PTCD/biliary stenting
- [ ] Hepatobiliary - TIPS
- [ ] Gastrointestinal - Enteral feeding tube insertion
- [ ] Gastrointestinal - Esophageal stenting
- [ ] Gastrointestinal - Colonic stenting
- [ ] Urological - Nephrostomy/ureteric stenting
- [ ] Women's/Men's health intervention - UFE/PAE/varicocele/pelvic congestion
- [ ] Percutaneous drainage - Thoracic
- [ ] Percutaneous drainage - Abdominal
- [ ] Vascular access - Central line/Portacath placement
- [ ] Biopsy - Percutaneous
- [ ] Biopsy - Transjugular liver biopsy
- [ ] Pediatric IR

Other: [ ] ________________________________

17. 17. In which areas have you seen a decreased demand for elective IR services? Please tick all that apply. *

Check all that apply.

- [ ] None
- [ ] Vascular - Aortic
- [ ] Vascular - Peripheral Limb
- [ ] Vascular - Venous
- [ ] Interventional oncology - Tumor ablation
- [ ] Interventional oncology - TACE/TAE
- [ ] Interventional oncology - TARE/Y90
- [ ] Dialysis access - Hemodialysis line and permanent access maintenance procedures
- [ ] Dialysis access - Peritoneal dialysis catheter insertion
- [ ] Dialysis access - Percutaneous AVF creation
- [ ] Hepatobiliary - PTCD/biliary stenting
- [ ] Hepatobiliary - TIPS
- [ ] Gastrointestinal - Enteral feeding tube insertion
- [ ] Gastrointestinal - Esophageal stenting
- [ ] Gastrointestinal - Colonic stenting
- [ ] Urological - Nephrostomy/ureteric stenting
- [ ] Women's/Men's health intervention - UFE/PAE/varicocele/pelvic congestion
- [ ] Percutaneous drainage - Thoracic
- [ ] Percutaneous drainage - Abdominal
- [ ] Vascular access - Central line/Portacath placement
- [ ] Biopsy - Percutaneous
- [ ] Biopsy - Transjugular liver biopsy
- [ ] Pediatric IR

Other: [ ] ________________________________

18. 18. Does your IR department have a day case recovery unit? *

Mark only one oval.

- ( ) Yes
- ( ) No

19. 19. If yes to question 18, is your day case recovery unit still operational?

Mark only one oval.

- ( ) Yes
- ( ) No
- ( ) Other: ________________________________

Skip to question 20

Section D: IR Clinic Visits

20. 20. Has there been any modifications to the outpatient clinics within your unit? *

Mark only one oval.

- No change
- All cancelled
- Triage of referrals and then review those eligible in person
- Video/telephone consultation for all
- Video/telephone consultation for triaged patients only

21. 21. Do Interventional Radiologists in your centre continue to have access to routine imaging slots (CT/MRI/US) in order to plan for potential procedures? *

Mark only one oval.

- Yes, readily available
- Yes, reduced availability
- No

Skip to question 22

Section E: Multi-Disciplinary Rounds

22. 22. Do Interventional Radiologists in your centre continue to participate in face-to-face multidisciplinary rounds? *

Mark only one oval.

- Yes
- No
- We do not normally have multidisciplinary rounds

23. 23. If you answered 'no' to question 22, how have you replaced face-to-face MDT rounds? *

Mark only one oval.

- Video conference/teleconference
- Limited core team attending, others dialing in as required
- Other: ______________________________

Skip to question 24

Section F: IR Training

Please complete the following section if your department has IR trainees. If your department does not have IR trainees please leave blank.

24. 24. How many IR trainees does your centre have?

Mark only one oval.

- No trainees
- 1
- 2
- 3
- 4
- 5
- 6
- 7
- 8
- 9
- 10
- 10+

25. 25. Has the average trainee case-load changed?

Mark only one oval.

- Yes, decreased by less than 25%
- Yes, decreased by 25-50%
- Yes, decreased by 51-75%
- Yes, decreased by over 75%
- Yes, increased
- No, unchanged

26. 26. Have your trainees been redeployed to other specialties?

Mark only one oval.

- Yes, to medical/surgical specialties
- Yes, to the emergency department
- Yes, to Critical Care Medicine (ICU)
- No, not been redeployed
- Other: ______________________

27. 27. Have provisions been made for virtual trainee teaching specifically for IR?

Mark only one oval.

- Yes
- No

28. 28. Have future attending positions for current IR trainees been affected as a result of the pandemic? Please tick all that apply.

Check all that apply.

- [ ] Yes, some positions have been cancelled
- [ ] Yes, some position start dates have been delayed
- [ ] No, future employment positions have not been affected

29. 29. Has employment of incoming IR trainees been affected as a result of the pandemic? Please tick all that apply.

Check all that apply.

- [ ] Yes, some individuals have cancelled their incoming training post
- [ ] Yes, some individuals have delayed the start date for their incoming training post
- [ ] Yes, the institution has cancelled some training posts
- [ ] Yes, the institution has delayed the start date for some training posts
- [ ] No, employment for incoming trainees has not been affected

Skip to question 30

Section G: COVID-19 Specific Questions

30. 30. Has your centre disseminated a PPE policy to members of your department specific to IR procedures? *

Mark only one oval.

- Yes
- No

31. 31. Are you able to follow the PPE policy? *

Mark only one oval.

- Yes
- No, PPE not readily available

32. 32. Do IRs in your centre participate in the provision of a dedicated central line service for COVID-19 patients? *

Mark only one oval.

- Yes
- No

33. 33. Are IRs in your centre performing procedures at the patient bedside in COVID suspected or positive patients where possible? *

Mark only one oval.

- Yes
- No

34. 34. Are elective/semi-urgent outpatients being tested for COVID-19 prior to treatment in your department? *

Mark only one oval.

- Yes
- No

35. 35. Has your department been linked to a COVID-19 outbreak at your institution? *

Mark only one oval.

- ( ) Yes
- ( ) No

This content is neither created nor endorsed by Google.

Google Forms
